# Large (≥3cm) thyroid nodules with benign cytology: Can Thyroid Imaging Reporting and Data System (TIRADS) help predict false-negative cytology?

**DOI:** 10.1371/journal.pone.0186242

**Published:** 2017-10-12

**Authors:** Se Jin Nam, Jin Young Kwak, Hee Jung Moon, Jung Hyun Yoon, Eun-Kyung Kim, Ja Seung Koo

**Affiliations:** 1 Department of Radiology, Severance Hospital, Research Institute of Radiological Science, Yonsei University, College of Medicine, Seoul, Korea; 2 Department of Pathology, Severance Hospital, Yonsei University, College of Medicine, Seoul, Korea; University of Toronto, CANADA

## Abstract

**Background:**

There is controversy about the accuracy of the fine-needle aspiration (FNA) cytology results in large sized thyroid nodules. Our aim was to evaluate the false-negative rate of FNA for large thyroid nodules and the usefulness of the Thyroid Imaging Reporting and Data System (TIRADS) in predicting false-negative cytology for large thyroid nodules with benign cytology.

**Methods:**

632 thyroid nodules larger than or equal to 3cm in size with subsequent benign cytology on US-guided FNA were included. US features of internal composition, echogenicity, margin, calcifications, and shape were evaluated, and nodules were classified according to TIRADS. TIRADS category 3 included nodules without any of the following suspicious features:solidity, hypoechogenicity or marked hypoechogenicity, microlobulated or irregular margins, microcalcifications, and taller-than-wide shape. Category 4a, 4b, 4c, and 5 were assigned to nodules showing one, two, three or four, or five suspicious US features, respectively. US features associated with malignancy for these lesions were analyzed and malignancy risk according to TIRADS was calculated.

**Results:**

Of the 632 lesions, 23 lesions(3.6%) were malignant and 609(96.4%) were benign, suggesting a 3.6% false-negative rate for FNA cytology. Of the 23 malignant lesions, final pathology was mainly follicular carcinoma minimally invasive(65.2%, 15/23) and the follicular variant of papillary carcinoma(26.1%, 6/23). The malignancy risks of categories 3, 4a, 4b, and 4c nodules were 0.9%, 4.6%, 10.0%, and 11.8%, respectively.

**Conclusion:**

Large thyroid nodules with benign cytology had a relatively high false-negative risk of 3.6% and TIRADS was helpful in predicting false-negative cytology for these lesions.

## Introduction

Fine-needle aspiration (FNA) has long been accepted as the most accurate method for evaluating thyroid nodules and selecting patients for thyroid surgery. The decision to perform FNA or clinical follow-up is based on clinical and ultrasonography (US) features and lesion size, according to current guidelines [1–4]. After FNA, patients with benign cytology are usually followed by clinical evaluation or US without repeated FNA, because there is a low possibility of malignancy (0–3%) [5]. However, for nodules with suspicious US features or increase in size during follow-up, even after benign cytology, repeat FNA is recommended to prevent delayed diagnosis of malignancy from possible false-negative FNA results [6,7]. Meanwhile, for large lesions, until now, the relationship between size and false-negative cytology remains controversial [8–13] and US features have yet to have an established role in predicting false-negative cytology for these large lesions.

The Thyroid Imaging Reporting and Data System (TIRADS) was first introduced by Horvath et al. for better communication between radiologists and physicians through a standardized reporting format [14] and originated from the Breast Imaging Reporting and Data System. Since its first introduction, several versions of TIRADS have been reported. The TIRADS by Kwak et al., which is based on the number of suspicious US features, is accurate for risk stratification and is very simple to use. Several recent studies reported that this TIRADS can help accurately stratify malignancy risk of thyroid nodules after FNA and can be easily applied in clinical practice owing to its simplicity [15–17]. However, this reporting system has not been applied to only large thyroid nodules with benign cytology, even when it could have been used in the continuous risk stratification of these nodules. Therefore, the purpose of this study was to figure out the false-negative cytology rate of large thyroid lesions and the criteria for selecting those for further work-up according to TIRADS.

## Materials and methods

The Institutional Review Board of Yonsei University College of Medicine, Severance Hospital, Seoul, Korea approved this retrospective study, and the requirement for informed consent was waived. Signed informed consent was obtained from all patients prior to biopsy or surgical procedures.

### Study population

By searching the FNA database of our institution from January 2010 to August 2014, we collected lesions larger than or equal to 3cm in size which were subsequently proven benign on US-guided FNA. Out of a total 1403 cases, those with the following conditions were included (n = 632): 1) lesions which underwent surgery (n = 164), 2) lesions that did not undergo surgery (n = 468; additionally diagnosed as benign or malignant at follow-up US-guided FNA (n = 97) or core needle biopsy (n = 1), alcohol injection along with shrinkage on follow-up US (n = 36), and with more than 1 year of follow-up US without change or with size decrease (n = 334). The mean follow-up period of 334 nodules was 764.7 ± 340.9 days (range, 365–1573 days). Those with the following conditions were excluded as the lesions were not precisely diagnosed (n = 771): 1) nondiagnostic or atypia on prior (n = 34) or follow-up US-guided FNA (n = 14) without surgery, 2) no more than 1 year of follow-up US (n = 723). Finally, a total 632 nodules in 632 patients were included in this study. The mean age of the patients was 49.5±14.0 years (range, 13–85 years). The mean size of the 632 nodules was 4.0±0.91 cm (range, 3–10 cm).

### US examination

During the study period, US examinations were performed by 10 radiologists with 1–16 years of experience, using a 5–12-MHz linear array transducer (iU22; Philips Medical Systems). Since 2006, our institutional registry has prospectively collected data on all patients with thyroid nodules who undergo US-guided FNAs at our institution. Nodule size was defined according to the largest diameter on US. Interpretation of the US features of all thyroid lesions was prospectively recorded according to internal composition, echogenicity, margin, calcifications and shape by the radiologist who performed thyroid US at the time of US-guided FNA.The internal composition was classified as solid or mixed, which included predominantly cystic (cystic portion >50%) or predominantly solid (cystic portion ≤50%) composition. Echogenicity was classified as hyper-, iso- or hypoechogenicity (when a nodule was hyper-, iso-, or hypoechoic compared with a normal thyroid gland), or marked hypoechogenicity (when a nodule was relatively hypoechoic compared with the surrounding strap muscle). Margins were classified as well-defined or not well-defined, which included microlobulated or irregular margins. Calcifications were classified as microcalcification (≤1mm in diameter; tiny, punctuate, hyperechoic foci, either with or without acoustic shadows) or no microcalcification which included macrocalcification or no calcification. Shape was classified as wider-than-tall or taller-than-wide (greater in the anteroposterior dimension than in the transverse dimension). A TIRADS category was assigned to each nodule based on the number of suspicious US features: solidity, hypoechogenicity or marked hypoechogenicity, microlobulated or irregular margins, microcalcifications, and taller-than-wide shape [15]. Thyroid nodules without suspicious features were classified as TIRADS category 3. Thyroid nodules with one, two, three or four, or five suspicious US features were classified as category 4a, 4b, 4c, or 5, respectively.

### US- guided fine-needle aspiration

US-guided FNA was performed by the same radiologist who performed real-time US. Free-hand US-guided FNA was performed with a 23-gauge needle attached to a 2-mL disposable plastic syringe. Each lesion was aspirated at least twice with a range of two to five passes. Samples obtained from the first passage were expelled on glass slides, smeared, and placed immediately in 95% alcohol for Papanicolaou staining. The remaining material in the syringe was rinsed in saline for cell-block processing. Cytopathologists were not present during biopsies. Cytopathologists specializing in thyroid pathology interpreted the slides obtained from US-guided FNA. The Bethesda System for reporting thyroid cytopathology was used in the classification of cytology reports [5].

### Standard reference

Thyroid nodules with malignant results at surgery or core needle biopsy were classified as malignant. Thyroid nodules with benign results at surgery, those with subsequent benign cytology and the ones with no change, or that decreased in size with subsequent benign cytology at follow-up US-guided FNA, and with no change or decrease in size during at least 12 months of follow-up US after benign results on US-guided FNA were classified as benign. For the cases with surgical pathology of follicular variant of papillary thyroid carcinoma (FVPTC) in study period, the pathology slide was reviewed retrospectively. And noninvasive encapsulated follicular variant of papillary thyroid carcinoma which has recently been reclassified to noninvasive follicular thyroid neoplasm with papillary-like nuclear features (NIFTP) was regarded as benign [18].

### Statistical analysis

The patient age and lesion size were compared between benign and malignant nodules using independent t-test. Gender (M/F ratio), US characteristics and TIRADS categories were compared between benign and malignant nodules using the Chi square or Fisher’s exact test. The malignancy risks of the thyroid nodules according to TIRADS were calculated and presented with percentages. According to the TIRADS category, the number of nodules recommended for surgery because of cytology-image discordance and diagnostic performances were assessed for all thyroid nodules and for nodules which underwent surgery respectively. Statistical analysis was performed with SPSS for Windows, version 20.0 (IBM Corporation, Armonk, NY, USA). A statistical difference was defined with a two-sided P value <0.05.

## Results

The frequency and distribution of cytological diagnosis in our institutions can be found in our previous study [16]. The outcome of the 632 thyroid nodules of this study and the corresponding diagnostic procedure is described in Table 1.

**Table 1:** Outcome of the 632 thyroid nodules of the study and the corresponding diagnostic procedure.

	No. of Nodules
Malignant	23
Surgery	22 (95.7)
Core needle biopsy without surgery*	1 (4.3)
Benign	609
Surgery	142 (23.3)
Repeat benign cytology	97 (15.9)
No change or decreased in size for at least 1 year of sonographic follow-up	370 (60.8)

Numbers in parentheses are percentages.

*Lymphoma

Of a total 632 lesions, 609 (96.4%) were benign and 23 (3.6%) were malignant suggesting a false-negative cytology of 3.6%. Of the 164 resected lesions, 142 (86.6%) were benign and 22 (13.4%) were malignant suggesting a false-negative cytology of 13.4%.

Surgery was indicated (n = 164) for the following conditions; increase in size during follow-up (n = 35), abnormality on follow-up FNA or core needle biopsy (n = 17), FDG uptake on PET-CT (n = 1), suspicious lateral neck lymph node on US (n = 1), toxic nodule (n = 3), patient request due to cosmetic issues (n = 47), compressive symptoms (n = 36), a concurrent malignant thyroid nodule (n = 18), suspicious physical examination results (n = 2), and concurrent surgery performed for another part of the neck (n = 4). Of 23 malignancies, 22 (95.7%) underwent surgery; 15 were follicular carcinoma minimally invasive, 6 were the follicular variant of papillary carcinoma, and 1 was a conventional papillary carcinoma. One case was a malignant lymphoma which was confirmed with core needle biopsy and treated with chemotherapy and radiation therapy. Of 609 benign nodules, 142 (23.3%) underwent surgery; 125 were adenomatous hyperplasia, 12 were follicular adenoma, 3 were NIFTP which reported FVPTC originally, 1 was granulomatous thyroiditis, and 1 was Hurthle cell adenoma.

**Fig 1:**
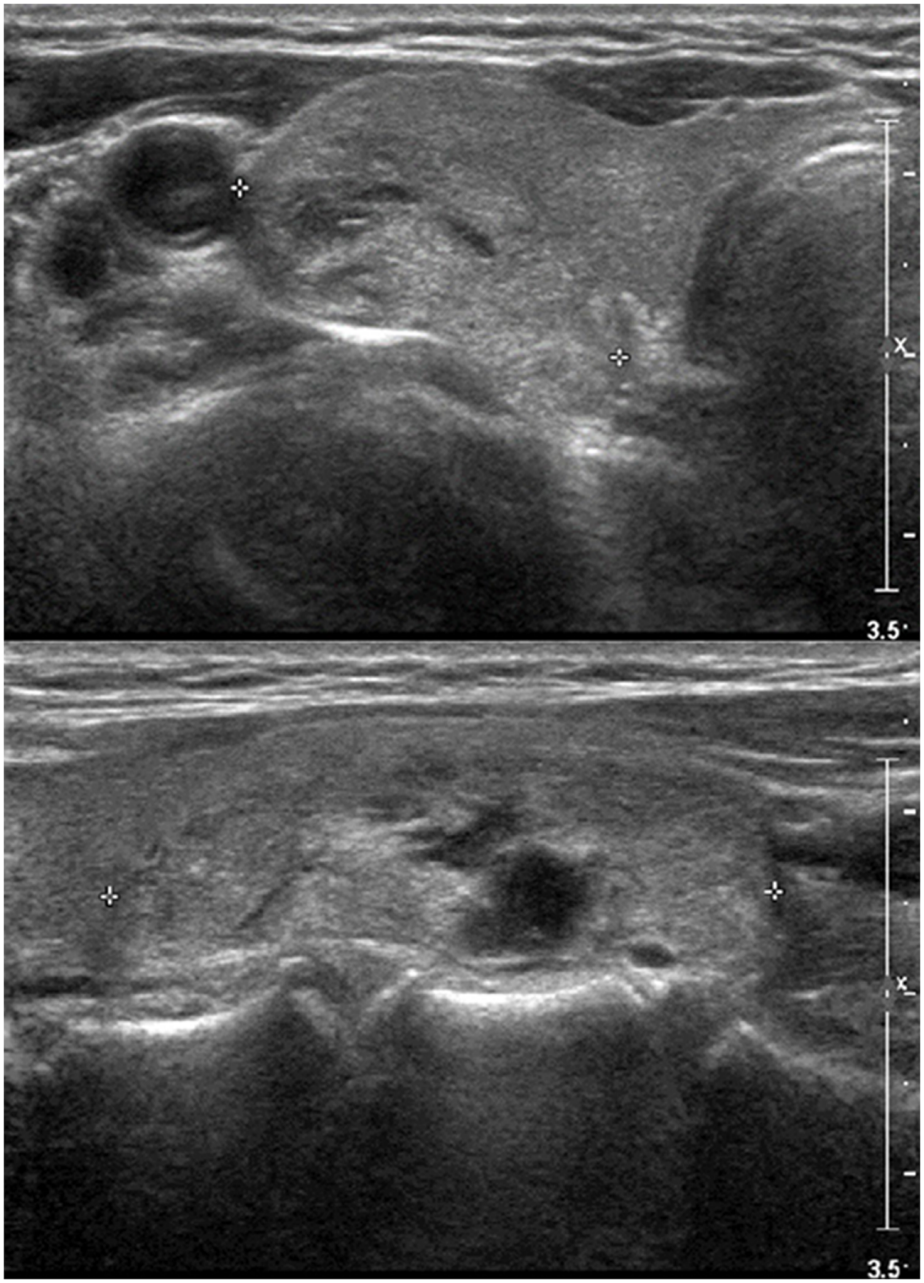
27-year-old female patient with 3.7cm-sized right thyroid nodule. On US (A: transverse image, B: longitudinal image), the lesion was well-defined, mixed isoechoic, predominantly solid, and wider-than tall without microcalcifications, suggesting TIRADS 3. FNA was performed due to its large size and the result was Bethesda class 2, benign. The patient underwent surgery due to contralateral thyroid malignancy and the final pathology was adenomatous hyperplasia.

**Fig 2:**
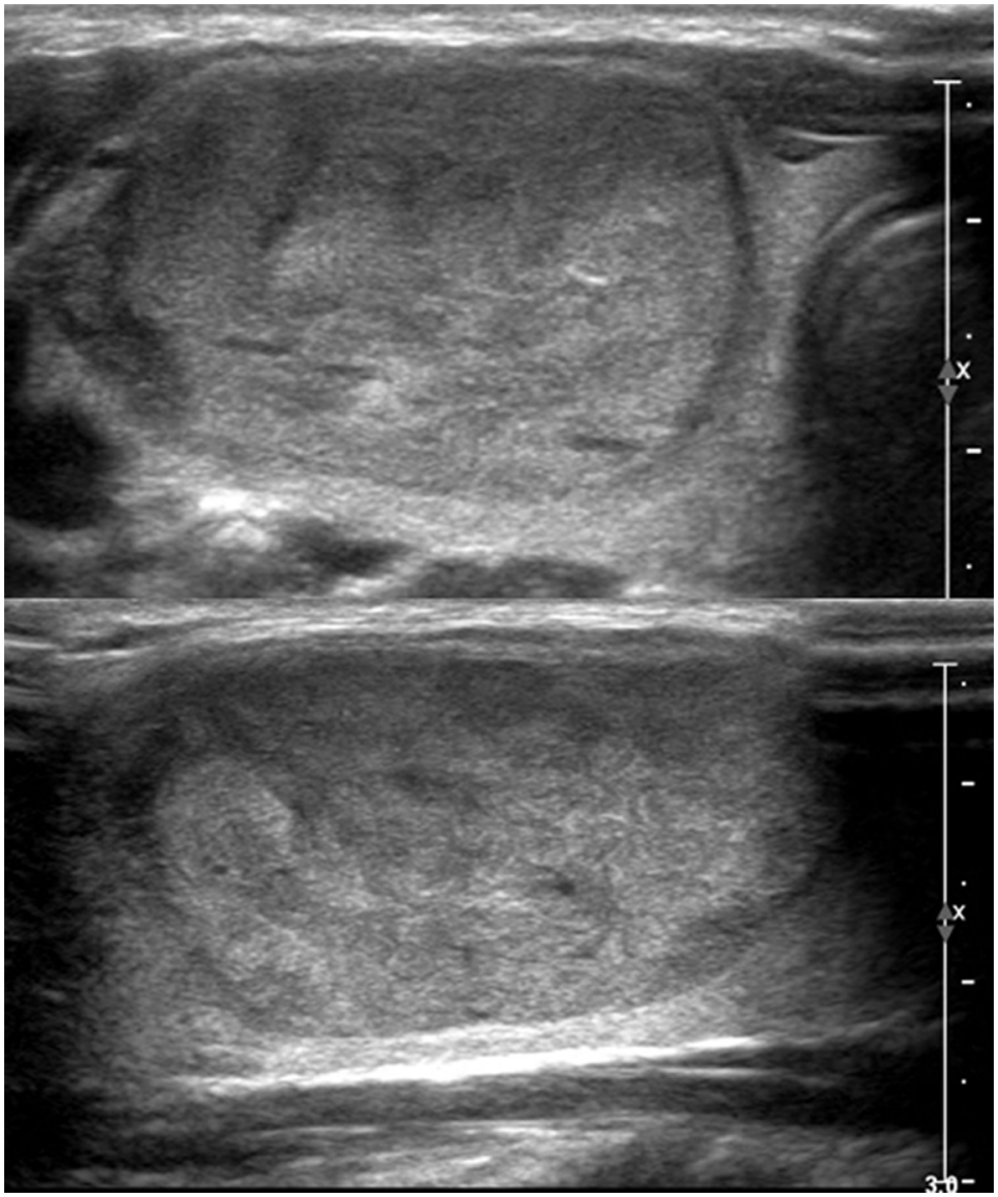
23-year-old female patient with 3.9cm-sized right thyroid nodule. On US (A: transverse image, B: longitudinal image), the lesion was well-defined, solid, isoechoic, and wider-than-tall without microcalcifications, suggesting TIRADS 4a. FNA was performed and the result was benign. The patient underwent surgery due to patients’ request and the final pathology was minimally invasive follicular carcinoma.

**Fig 3:**
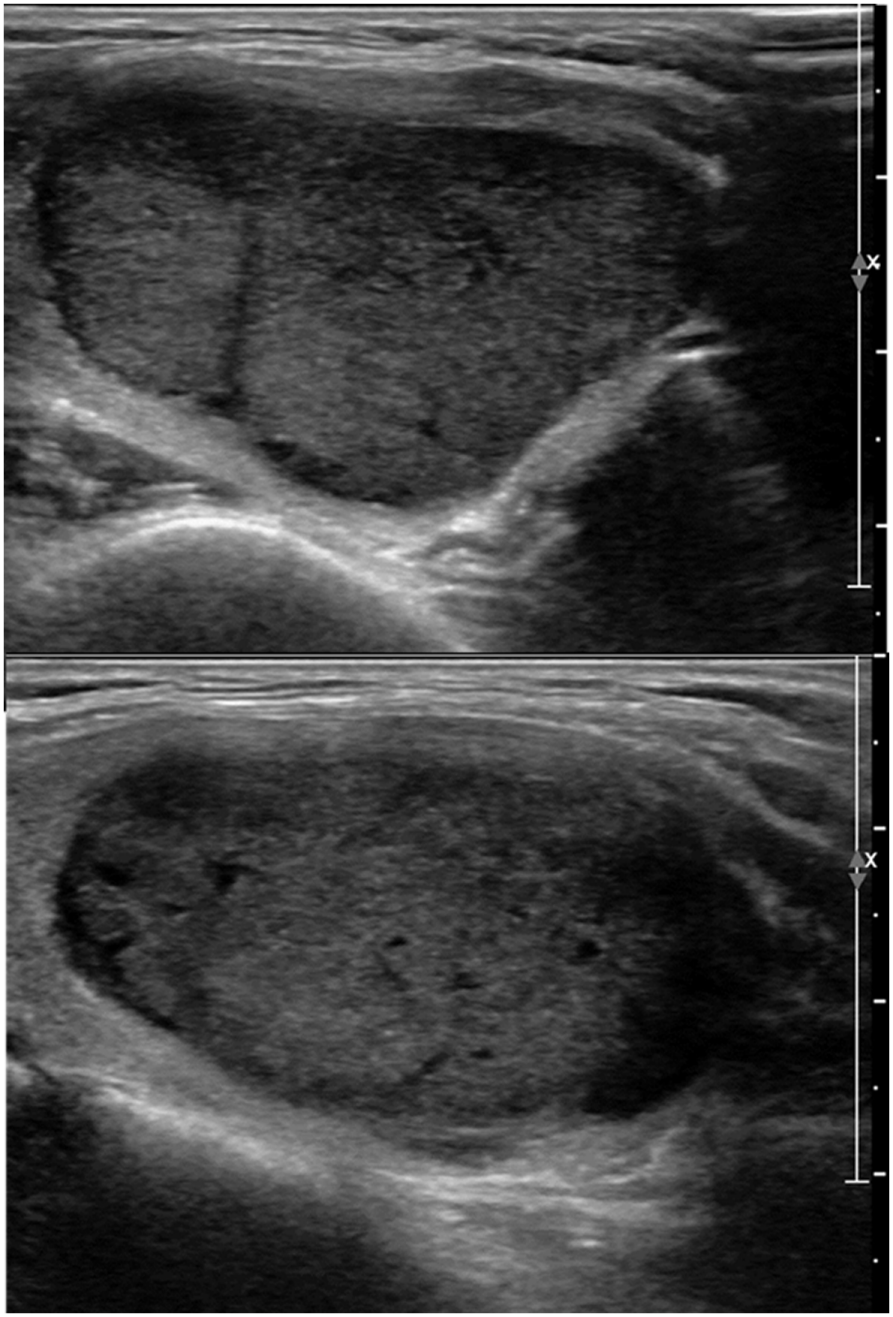
43-year-old female patient with 4.2cm-sized right thyroid nodule. On US (A: transverse image, B: longitudinal image), the lesion was well-defined, solid, marked hypoechoic, and wider-than-tall without microcalcifications, suggesting TIRADS category 4b. The FNA result was Bethesda class 2, benign. The patient underwent surgery due to contralateral thyroid malignancy and the final pathology was the follicular variant of papillary thyroid carcinoma.

When comparing US features of the 609 benign and 23 malignant lesions, solid composition (*P*<0.001), hypo- or marked hypoechogenicity (*P* = 0.001), and higher TIRADS category (*P*<0.001) were more frequently seen in malignant nodules than benign nodules. The malignancy risk increased as the TIRADS category increased. The malignancy risks of category 3, 4a, 4b and 4c were 0.9%, 4.6%, 10.0% and 11.8%, respectively. For the 164 cases with surgery, solid composition (*P* = 0.003), hypo- or marked hypoechogenicity (*P* = 0.003), and higher TIRADS category (*P* = 0.003) were more frequently seen in malignant nodule, same as all 632 lesions. The malignancy risks of category 3,4a,4b and 4c were 3.8, 18.9, 25.8 and 50.0%, respectively (Table 2). Representative examples are shown in Figs 1–3.

**Table 2:** Association between thyroid malignancy and various US features of all cases and surgery cases.

Parameter	All cases (n = 632)	Benign Nodules (n = 609)	Malignant Nodules (n = 23)	P value	Surgery cases (n = 164)	Benign Nodules (n = 142)	Malignant Nodules (n = 22)	P value
Age (y)		49.7±14.0	44.6±13.3	0.090^a^		48.4±14.3	43.6±12.7	0.139^a^
Gender (M/F ratio)		101/508	3/23	1.000^b^		19/123	3/19	1.000^b^
Lesion size (cm)		4.0±0.89	4.6±1.30	0.047^a^		4.4±1.19	4.6±1.28	0.512^a^
US features								
Composition				<0.001^b^				0.003^b^
Solid	231	214 (35.1)	17 (73.9)		71	55 (38.7)	16 (72.7)	
Mixed	401	395 (64.9)	6 (26.1)		93	87 (61.3)	6 (27.3)	
Echogenicity				0.001^b^				0.003^b^
Hypo-/Marked hypo-	152	140 (23.0)	12 (52.2)		40	29 (20.4)	11 (50.0)	
Iso- or Hyper-	480	469 (77.0)	11 (47.8)		124	113 (79.6)	11 (50.0)	
Margin				0.202^b^				0.518^b^
Not well-defined	23	21 (3.4)	2 (8.7)		5	4 (2.8)	1 (4.5)	
Well-defined	609	588 (96.6)	21 (91.3)		159	138 (97.2)	21 (95.5)	
Calcification				0.529^b^				0.585^b^
Microcalcification	20	19 (3.1)	1 (4.3)		6	5 (3.5)	1 (4.5)	
No microcalcification	612	590 (96.9)	22 (95.7)		158	137 (96.5)	21 (95.5)	
Shape				0.230^b^				0.134^b^
Taller-than-wide	7	6 (1.0)	1 (4.3)		1	0 (0)	1 (4.5)	
Wider-than-tall	625	603 (99.0)	22 (95.7)		163	142 (100)	21 (95.5)	
TIRADS category				<0.001^b^				0.003^b^
3	319	316 (51.9)	3 (13.0)		78	75 (52.8)	3 (13.6)	
4a	216	206 (33.8)	10 (43.5)		53	43 (30.3)	10 (45.5)	
4b	80	72 (11.8)	8 (34.8)		31	23 (16.2)	8 (36.4)	
4c	17	15 (2.5)	2 (8.7)		2	1 (0.7)	1 (4.5)	

Numbers in parentheses are percentages.

^a^ Independent t-test

^b^ Chi square or Fisher’s exact test

The number of nodules recommended for surgery because of cytology-image discordance and the diagnostic performances including sensitivity and positive predictive value (PPV) according to TIRADS category for all and surgery cases are shown in Table 3. If all large thyroid nodules with benign cytology had been recommended for surgery, no thyroid malignancies would have been missed, suggesting a sensitivity of 100% and a PPV of 3.6%. For nodules with TIRADS scores greater than or equal to 4a, the number of lesions recommended for surgery would have decreased to less than half (49.5%, 313 of 632), while the three malignancies (13.0%, three of 23) would have been missed, suggesting a sensitivity of 87.0% and a PPV of 6.4%. For only surgery cases, the false-negative rate and sensitivity were not different although the PPV increases increased.

**Table 3:** Diagnostic performance according the TIRADS category of all cases and surgery cases.

TIRADS category	All cases (n = 632)						Surgery cases (n = 164)					
	Nodules recommended for surgery*	TP	FP	FN	SEN (%,95%CI)	PPV (%,95%CI)	Nodules underwent surgery	TP	FP	FN	SEN (%,95%CI)	PPV (%,95%CI)
3,4a,4b,4c	632 (100)	23	609	0 (0)	100 (85.2–100)	3.6 (2.3–5.4)	164 (100)	22	142	0 (0)	100 (84.6–100)	13.4 (8.6–19.6)
4a,4b,4c	313 (49.5)	20	293	3 (13.0)	87.0 (66.4–97.2)	6.4 (5.4–7.5)	86 (52.4)	19	67	3 (13.6)	86.4 (65.1–97.1)	22.1 (18.2–26.5)
4b,4c	97 (15.3)	10	87	13 (56.5)	43.5 (23.2–65.5)	10.3 (6.5–16.0)	33 (20.1)	9	24	13 (59.1)	40.9 (20.7–63.7)	27.3 (16.8–41.1)
4c	17 (2.7)	2	15	21 (91.3)	8.7 (1.1–28.0)	11.8 (3.2–35.5)	2 (1.2)	1	1	21 (95.4)	4.6 (0.1–22.8)	50 (6.1–93.9)

Numbers in parentheses are percentages and 95% confidence interval

TP: true-positive, FP: false-positive, FN: false-negative, SEN: sensitivity, PPV: positive predictive value

*Number of surgeries that would have been performed had the TIRADS category been applied to the overall sample of nodules as cytology-image discordance

## Discussion

Regarding the accuracy of benign cytology of large thyroid nodules, there has been no consensus on whether large size is a risk factor for false-negative FNA and on how to subsequently manage these lesions [8–13, 19–26]. The reason for this dissensus is due to conflicting data and recommendations from prior studies. We reviewed the data of nodules with histologic evaluation from 14 previous studies published in English-language literature [8–13, 19–26]. to allow accurate comparisons, and the range and interpretation of these nodules remained quite broad (Table 4). Six studies recommended surgery [9–11,13,19,25] or repeat FNA [10] for large thyroid nodules even after benign FNA results because of a high false-negative rate (FNR) of 7.7–25.0%. In contrast to those studies, the remainder studies mentioned that large size itself should not be an indication for surgery because the FNR was within an acceptable range of 0.7–3.6% [20, 22, 24, 26] or not different from that of smaller nodules despite having a high FNR of 4.3–15.0% [8, 12, 21, 23]. In the present study, the FNR of large thyroid nodules with benign cytology was 3.6% (23 of all 632 nodules including follow-up nodules) to 13.4% (22 of 164 resected nodules) which was higher than the Bethesda recommendation of 0–3%. Therefore, our result supported the claim that benign cytology results of large thyroid nodules can be inaccurate, and consequently, that US follow-up is insufficient for these large lesions.

**Table 4:** Published literature review of false-negative fine-needle aspiration rates in large thyroid nodules with histology.

Author (Ref. No.)	Year	Size criteria	Number of nodules with benign FNA*	Number of nodules with malignancy	False-negative rate (%)	Recommendation
Meko and Norton (19)	1995	≥3	16	4	25.0	Surgery
Carrillo (10)	2000	≥4	35	7	20.0	Close follow up or repeat FNA
		<4	39	2	5.1	
McCoy (11)	2007	≥4	71	9	12.7	Surgery
Giles (13)	2015	≥3	240	28	11.7	Surgery
		≥4	146	16	11.0	
Wharry (25)	2014	≥4	125	13	10.4	Surgery
Pinchot (9)	2009	≥4	52	4	7.7	Surgery
Albuja-Cruz (8)	2013	≥4	113	17	15	Not indication for surgery
		<4	209	25	12	
Mehanna (12)	2013	≥3	55	6	10.9	Not indication for surgery
		<3	33	2	6.1	
Shrestha (23)	2012	≥4	98	7	7.1	Not indication for surgery
		<4	319	22	6.9	
Kuru (21)	2010	≥4	98	4	4.3	N/A†
		<4	319	4	1.3	
Rosario (20)	2009	≥4	84	3	3.6	Not indication for surgery
Yoon (26)	2011	≥3	112 (558^a^)	2	1.8 (0.4^b^)	Not indication for surgery
Raj (24)	2012	≥4	118	1	0.8	Not indication for surgery
Porterfield (22)	2008	≥3	145 (694^a^)	1	0.7 (0.1^b^)	Not indication for surgery
Present study		≥3	164 (632^a^)	22	13.4 (3.6^b^)	Surgery considering US features

*FNA, fine-needle aspiration

^†^N/A not applicable

^a^ Number of nodules with benign FNA including follow-up cases without surgery

^b^ False-negative rate including follow-up cases without surgery

In analyzing the pathologic outcomes of these false-negative cytology lesions of large thyroid nodules, we were able to observe that thyroid malignancy with follicular morphologic features including FVPTC accounted for the majority (91.3%, 21 of 23) of malignancies in our study, even after 3 FVPTC are recategorization as NIFTP according to the recent nomenclature revision. [18]. This observation was not surprising. Follicular carcinomas containing the macrofollicular pattern with abundant background colloid are known to be easily mistaken as benign adenomatoid colloid nodules on cytology which may be the part of the cause for false-negative results [27]. The FVPTC also has been described as the most common histologic malignancy diagnosed after cytologically benign FNA [8, 12, 28], and when the pathognomonic nuclear features of the FVPTC are only focally present, the diagnosis can easily be missed. Our study result corresponds with these prior concepts.

We evaluated whether sonographic features can help predict false-negative cytology for the large thyroid lesions with benign cytology, focusing on the TIRADS classification. The TIRADS developed by Kwak et al [15] enables stratification of thyroid nodules according to malignancy risk. TIRADS category 3 is assigned to thyroid nodules without suspicious features, and categories 4a, 4b, 4c, and 5 are assigned to nodules with one, two, three or four, or five suspicious features, respectively [15]. Recently, Moon et al. reported on the malignancy risks of nodules with benign cytology according to the TIRADS classification and the malignancy risks of TIRADS 3, 4a, 4b, 4c and 5 were 0.7%, 1.2%, 0.7%, 9.8% and 22.2% respectively [16]. Moon et al. concluded that benign nodules with more than 3 suspicious US features (TIRADS 4c and 5) should undergo repeat FNA [16]. In our study population of large (≥3cm) thyroid nodules with benign cytology, the malignancy risks of categories 3, 4a, 4b, and 4c nodules were much higher at 0.9%, 4.6%, 10.0%, and 11.8%, respectively. The 0.9% malignancy risk of category 3 was within the 0%-3% range, which is the risk range for recommending clinical follow-up in benign nodules with the Bethesda system [5]. Thus, US follow-up can be sufficient for large nodules with benign cytology if the nodules have no suspicious US features. However, for lesions with TIRADS scores greater than or equal to 4a, further work-up should be performed due to the high risk of malignancy. In this study, the malignancy risk for each category was higher for operated cases than all nodules and this discrepancy also arose because surgery was performed more selectively on nodules with suspicious physical examinations, repeat cytology abnormalities, or growing lesions.

Wharry et al. recently insisted on thyroid lobectomy for all nodules ≥4cm while reporting a high malignancy rate of 22% and a high false-negative rate of benign cytology of 10.4% [25]. However, as in our results shown in Table 3, if all large benign lesions were recommended for surgery, unnecessary surgeries would be performed although no malignancies would be missed. For nodules with TIRADS scores greater than or equal to 4a regarded as cytology-image discordance, the number of lesions recommended for surgery would have decreased to less than half the entire cases (49.5%, 313 of 632), while the three malignancies (13.0%, three of 23) would have been missed.

There is controversy on how to manage large thyroid nodules with benign cytology. Repeat FNA is usually chosen as the next diagnostic method for possible false-negative cytologic lesions, but might not be adequate for the management for large benign thyroid nodules because the majority of pathology in this category is follicular carcinoma or the FVPTC which is often mistaken by FNA [8, 12, 27, 28]. Thyroid core needle biopsy is emerging as a diagnostic method for thyroid disease and has been increasingly used when making conclusive diagnoses for nodules with nondiagnostic, or indeterminate cytologic results [29, 30] and in the diagnosis of follicular neoplasms [31, 32]. However, there is still lack of evidence on whether core needle biopsy is more advantageous in predicting malignancy in the preoperative differential diagnosis of follicular neoplasm, especially considering the major pathology of false-negative FNA in large thyroid nodules [33]. The role of core needle biopsy for this disease group should be established with further study.

Recently, Lee et al. published a study on the cost-effectiveness of diagnostic lobectomy versus observation of thyroid nodules >4cm with benign cytology after FNA. Lee et al. concluded that thyroid lobectomy is associated with improved outcomes at an acceptable cost in the management of large benign thyroid nodules [34]. Even though diagnostic lobectomy is cost-effective, routine surgery should be performed carefully for select patients due to possible thyroidectomy-related complications such as recurrent laryngeal nerve injury, postoperative bleeding, infection, and cosmetic problems such as skin scars. According to our results, US features with the TIRADS classification should be predominantly considered to select which patients should undergo diagnostic lobectomy. US features of follicular carcinoma or FVPTC are known to be underestimated when analyzed with published sonographic criteria because follicular carcinoma and FVPTC show a more benign appearance such as regular solid iso- or mild hypoechoic nodules rather than the classic suspicious features of markedly hypoechogenicity or microcalcifications [35, 36]. The majority of these lesions show an average large size and mainly solid echogenicity [36, 37]. In this context, we suggest that, the TIRADS classification system by Kwak et al. which includes ‘solidity’ as a suspicious feature can be used to stratify the malignancy risk of these large benign thyroid nodules.

The limitations of our study are as follows. First, due to the retrospective nature of our study, a patient selection bias might have occurred. Particularly, during the study period, more than 50% (771/1403) of the large nodules obtained benign results on FNA were excluded from the analysis because the lesions were not precisely identified. Although possible concern for selection bias, for the more reliable results, we think it would be more appropriate to exclude them from the analysis and hope that further study will be possible through a longer follow-up period for these lesions. Second, although our study was a single center study, 10 board-certified radiologists performed the US examinations and US-guided FNAs. Thus, intra- and interobserver variabilities in interpreting the US results exist. However, previous published data from our institution revealed more than a moderate degree of agreement in US assessment of thyroid nodules among experienced radiologists [38] and other studies have also shown that the diagnostic performances of malignant stratification using TIRADS categories are comparable regardless of the performer’s level of experience [39, 40]. These results support our opinion that any bias due to having 10 different US operators participate in the present study did not significantly affect our study results nor their interpretation. Third, a large portion of the benign thyroid nodules that did not undergo surgery were assumed to be benign, but the possibility of malignancy cannot be completely excluded even with repeat benign cytology. Thus, this might affect an underestimation of the malignancy rate in this study.

In conclusion, large thyroid nodules with benign cytology results had a relatively high false-negative risk of 3.6% and TIRADS was helpful in predicting false-negative cytology. If large thyroid nodules with benign cytology have any suspicious US features, additional work-up such as surgery should be recommended.
